# The impact of HIV infection and socioeconomic factors on the incidence of gonorrhea: A county-level, US-wide analysis

**DOI:** 10.1371/journal.pone.0183938

**Published:** 2017-09-01

**Authors:** Nikolaos Andreatos, Christos Grigoras, Fadi Shehadeh, Elina Eleftheria Pliakos, Georgianna Stoukides, Jenna Port, Myrto Eleni Flokas, Eleftherios Mylonakis

**Affiliations:** Infectious Diseases Division, Warren Alpert Medical School of Brown University, Rhode Island Hospital, Providence, Rhode Island, United States of America; Emory University School of Medicine, UNITED STATES

## Abstract

**Background:**

Gonorrhea is the second most commonly reported identifiable disease in the United States (U.S.). Importantly, more than 25% of gonorrheal infections demonstrate antibiotic resistance, leading the Centers for Disease Control and Prevention (CDC) to classify gonorrhea as an “urgent threat”.

**Methods:**

We examined the association of gonorrhea infection rates with the incidence of HIV and socioeconomic factors. A county-level multivariable model was then constructed.

**Results:**

Multivariable analysis demonstrated that HIV incidence [Coefficient (Coeff): 1.26, 95% Confidence Interval (CI): 0.86, 1.66, P<0.001] exhibited the most powerful independent association with the incidence of gonorrhea and predicted 40% of the observed variation in gonorrhea infection rates. Sociodemographic factors like county urban ranking (Coeff: 0.12, 95% CI: 0.03, 0.20, P = 0.005), percentage of women (Coeff: 0.41, 95% CI: 0.28, 0.53, P<0.001) and percentage of individuals under the poverty line (Coeff: 0.45, 95% CI: 0.32, 0.57, P<0.001) exerted a secondary impact. A regression model that incorporated these variables predicted 56% of the observed variation in gonorrhea incidence (Pmodel<0.001, R^2^ model = 0.56).

**Conclusions:**

Gonorrhea and HIV infection exhibited a powerful correlation thus emphasizing the benefits of comprehensive screening for sexually transmitted infections (STIs) and the value of pre-exposure prophylaxis for HIV among patients visiting an STI clinic. Furthermore, sociodemographic factors also impacted gonorrhea incidence, thus suggesting another possible focus for public health initiatives.

## Introduction

According to the Centers for Disease Control and Prevention (CDC), gonorrhea is the second most commonly reported identifiable disease in the United States (U.S.) [1, 2]. At the same time, the CDC estimates that more than 25% of new *Neisseria gonorrhoeae* infections demonstrate antibiotic resistance and has classified gonorrhea as an “urgent threat” [3]. Importantly, inadequately treated infections often result in serious complications and long-term morbidity, while infected individuals may remain asymptomatic for extensive periods thus facilitating disease transmission [4]. Moreover, gonococcal infections promote the transmission of HIV thus exerting a disproportionately severe, indirect impact on the reproductive health of the community [5, 6].

Given the scarcity of available public health resources, effective interventions must be targeted at high-risk populations. Such a strategy would be particularly effective against gonorrhea, as wide variations in disease incidence exist in different sociodemographic groups [7, 8]. Even though several studies have examined the influence of sociodemographic variables on the incidence and outcome of gonorrhea [4, 7, 9–13], little information exists on the precise impact and relative importance of these factors and the association of gonorrhea infection rates with the incidence of HIV on a national scale. In this cross-sectional study, we utilized nationwide data from the National Center for HIV/AIDS, Viral Hepatitis, Sexually Transmitted Diseases, and Tuberculosis Prevention (NCHHSTP) Atlas database [14] and the U.S. Census Bureau [15], in order to examine the association of gonorrhea with socioeconomic variables and HIV incidence. We then constructed a multivariable model of gonorrhea incidence on the basis of the identified variables.

## Methods

### Gonorrhea and HIV infection incidence rates

To estimate the gonorrhea and HIV infection rates, we extracted all relevant information from the NCHHSTP Atlas database of the CDC [14]. Annual incidence rates for the entire U.S. population were calculated for the 2010–2014 period, the 5 most recent years with available data. We also estimated the corresponding average gonorrhea and HIV incidence rates [and 95% Poisson confidence intervals (CIs)] over the 2010–2014 time-period, for each state and county with available data. The District of Columbia and Puerto Rico were each considered as a state for the purpose of our analysis and all average incidence rates were adjusted for county population, before being incorporated into the county-level analysis.

### Socioeconomic variables

Socioeconomic information for each county was obtained from the 2010–2014 American Community Survey (ACS) 5-year estimates dataset [15]. This dataset and the aforementioned time-horizon were selected due to their proven reliability in estimating demographic variables from U.S. counties [16]. Information on the following independent variables was extracted and included in our analysis: a) Percentage of individuals living under the poverty line, b) Percentage of individuals of Black, Hispanic or White race/ethnicity, c) Percentage of women, and, d) Urban *vs*. rural ranking of counties.

### Statistical analysis

We performed univariable linear regression to examine the association of population-adjusted, average gonorrhea incidence rates at the county level with possible predictive variables, specifically: population-adjusted, average HIV diagnosis rate, percentage of individuals living under the poverty line, population distribution according to race and gender and county urban *vs*. rural ranking. A multivariable linear regression model was then constructed by incorporating variables that were significantly associated with gonorrhea incidence in univariable analysis and were judged to be important from a biomedical perspective.

The regression coefficient of each variable (Coeff), the corresponding P value and the coefficient of determination (R^2^), indicating the proportion of variance in the dependent variable that stemmed from the independent variable, were calculated in each case. To facilitate comparisons, the statistical distribution of each variable was appropriately adjusted, so as to produce a mean of 0 and a standard deviation (SD) of 1 (Z-score). We employed Belsley’s test to examine collinearity between independent variables [17]. Statistical significance was defined as P<0.05. All data processing and statistical analyses were performed using STATA v.14 software (StataCorp LP, College Station, TX). Incidence rate choropleth maps were created using the Quantum Geographic Information System (QGIS) [18] and a hotspot analysis map was generated with the aid of the ArcGIS online platform (Environmental Systems Research Institute, Redlands CA) [19].

## Results and discussion

**Fig 1:**
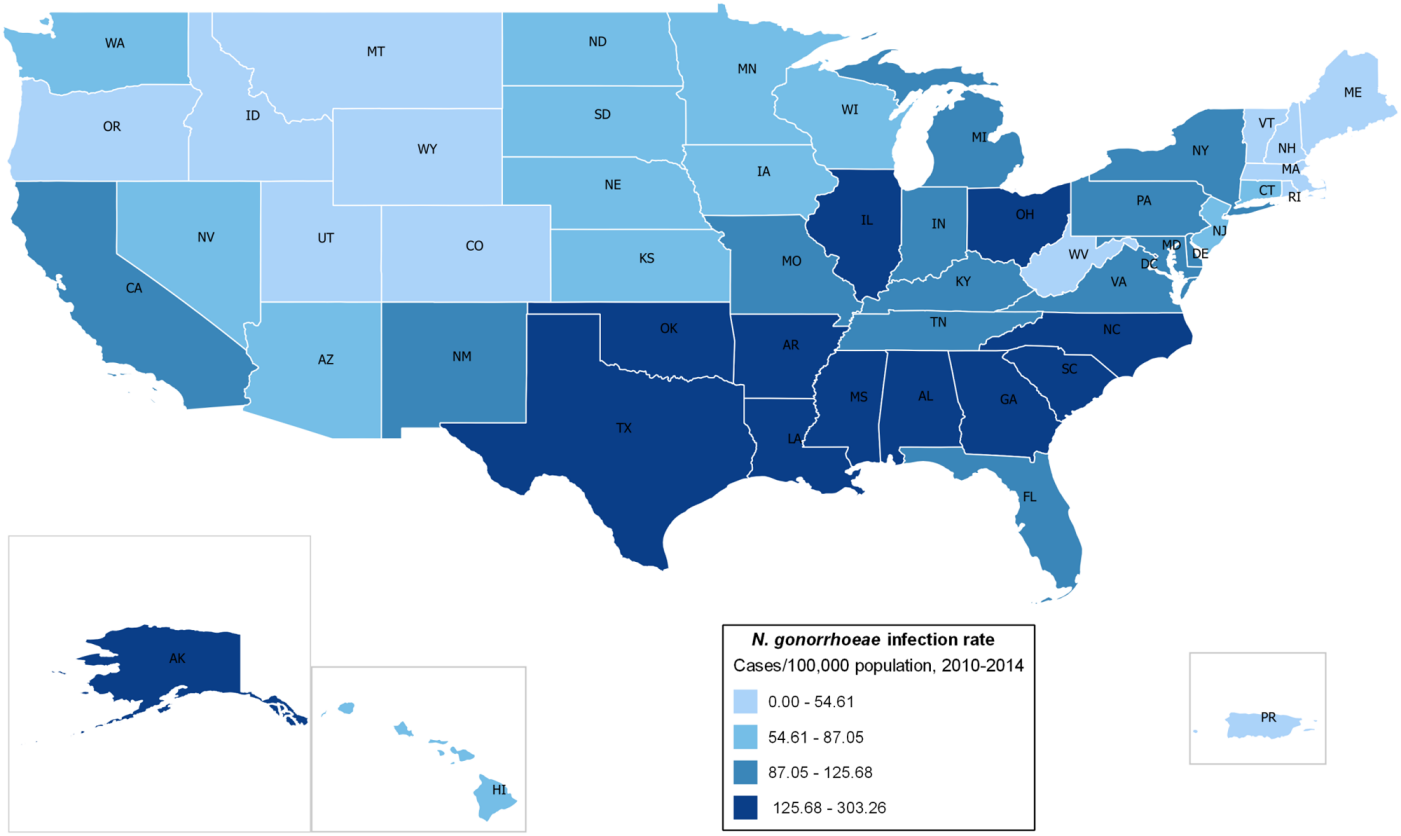
*N*. *gonorrhoeae* infection rates across the United States. Choropleth Map of Gonorrhea Incidence in the United States, 2010–2014.

**Fig 2:**
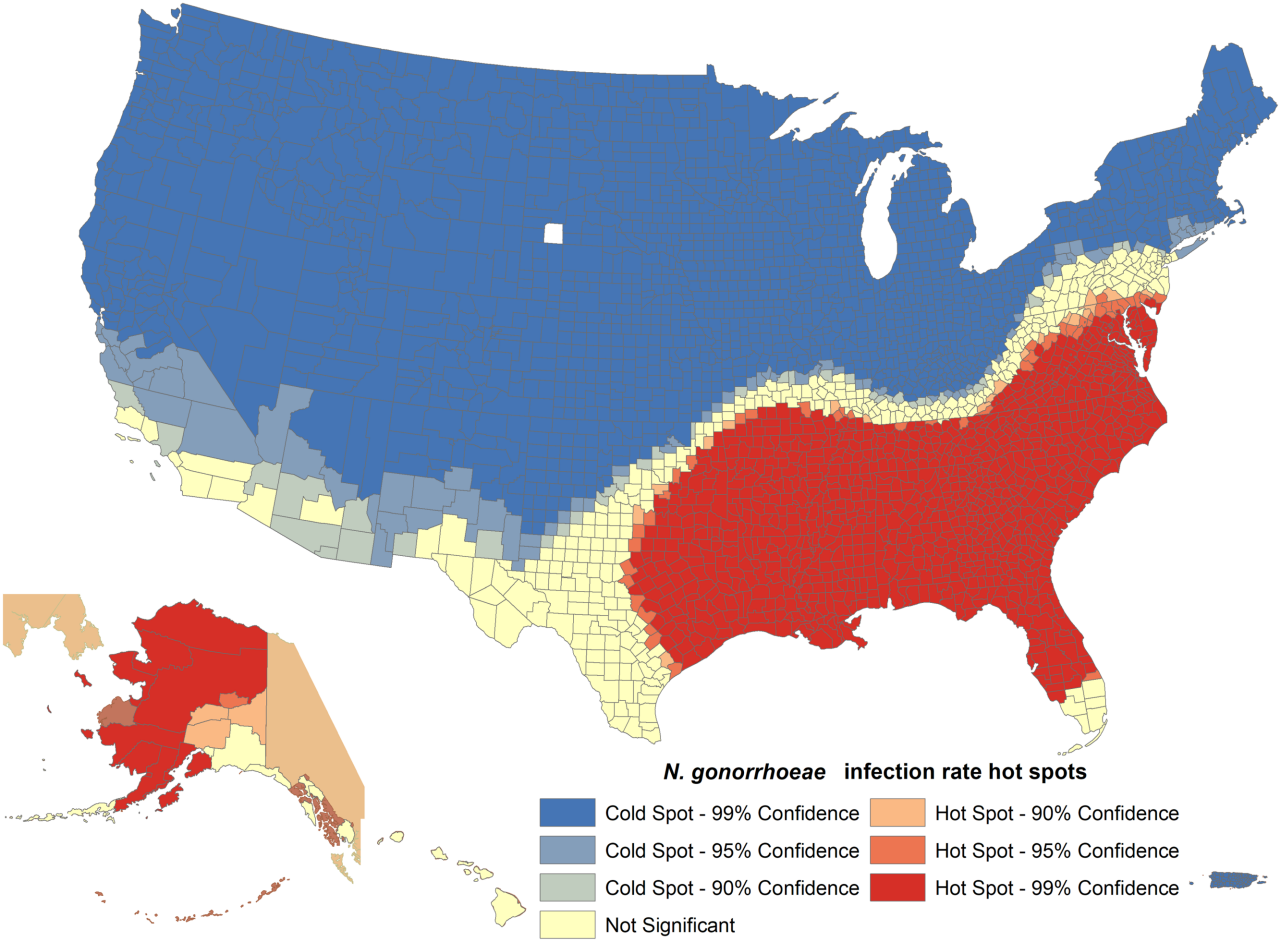
*N*. *gonorrhoeae* infection hotspots in the United States. Gonorrhea Incidence Hotspot Analysis in the United States, 2010–2014.

A total of 1,638,863 *N*. *gonorrhoeae* infections were reported in 3,220 counties or county-equivalents (from the U.S., Puerto Rico, the U.S. Virgin Islands and Guam) between 2010 and 2014. The adjusted average gonorrhea incidence rate was 103.30 cases per 100,000 people (95% CI: 103.14, 103.45) and the incidence of gonorrhea increased from 100.19 cases per 100,000 people in 2010 to 109.79 cases per 100,000 people in 2014. The District of Columbia [303.26 cases per 100,000 people (95% CI: 297.21, 309.40)], Mississippi [197.17 cases per 100,000 people (95% CI: 194.92, 199.43)] and Louisiana [194.10 cases per 100,000 people (95% CI: 192.31, 195.91)] had the highest reported rates, while New Hampshire [11.74 cases per 100,000 people (95% CI: 10.92, 12.59)], Wyoming [10.86 cases per 100,000 people (95% CI: 9.69, 12.13)] and Puerto Rico [9.90 cases per 100,000 people (95% CI: 9.45, 10.37)] had the lowest rates of *N*. *gonorrhoeae* infection. Overall, the highest incidence rates were concentrated in the Southern states (Figs 1 and 2, Table 1).

**Table 1:** *N*. *gonorrhoeae* infection rates by state, 2010–2014.

State	Reported Cases	Incidence Rate (Cases/100,000 Population)	95% Confidence Interval	
**DC**	9,556	303.26	297.21	309.40
**MS**	29,411	197.17	194.92	199.43
**LA**	44,593	194.10	192.31	195.91
**AL**	42,296	175.70	174.03	177.39
**SC**	39,261	166.48	164.84	168.14
**NC**	73,962	152.01	150.92	153.11
**AR**	22,309	151.46	149.48	153.46
**AK**	5,433	149.35	145.40	153.37
**GA**	70,526	142.70	141.65	143.75
**OH**	79,702	137.92	136.96	138.88
**IL**	83,397	129.64	128.76	130.53
**OK**	24,367	127.83	126.23	129.45
**TX**	164,083	126.37	125.76	126.98
**MO**	37,783	125.45	124.18	126.72
**MI**	59,341	120.06	119.10	121.03
**TN**	38,459	119.43	118.24	120.63
**DE**	5,405	118.11	114.98	121.30
**MD**	31,651	107.77	106.58	108.96
**PA**	68,627	107.63	106.82	108.44
**IN**	34,816	106.52	105.40	107.64
**VI**	554	105.28	96.69	114.42
**FL**	101,076	104.90	104.25	105.55
**NY**	102,270	104.59	103.95	105.24
**KY**	21,817	99.69	98.37	101.02
**CA**	171,110	90.22	89.79	90.64
**VA**	36,002	88.16	87.25	89.07
**NM**	9,113	87.65	85.86	89.47
**NV**	11,893	86.46	84.91	88.03
**AZ**	27,171	83.14	82.15	84.13
**SD**	3,453	82.93	80.19	85.74
**WI**	23,255	81.29	80.25	82.34
**KS**	11,250	78.12	76.68	79.57
**NJ**	34,203	77.20	76.38	78.02
**NE**	6,785	73.24	71.51	75.00
**CT**	11,899	66.28	65.10	67.48
**HI**	3,994	57.56	55.79	59.37
**IA**	8,842	57.53	56.33	58.74
**WA**	19,429	56.48	55.69	57.28
**ND**	1,976	56.34	53.88	58.88
**GU**	448	55.98	50.91	61.41
**MN**	14,739	54.84	53.96	55.73
**CO**	13,959	53.91	53.02	54.81
**WV**	4,110	44.31	42.97	45.69
**MA**	14,077	42.40	41.70	43.11
**RI**	2,199	41.84	40.10	43.62
**OR**	8,076	41.49	40.59	42.40
**UT**	3,456	24.26	23.46	25.08
**ME**	1,372	20.66	19.58	21.78
**MT**	953	18.97	17.78	20.21
**ID**	1,130	14.17	13.36	15.02
**VT**	386	12.33	11.13	13.62
**NH**	775	11.74	10.92	12.59
**WY**	312	10.86	9.69	12.13
**PR**	1,801	9.90	9.45	10.37

Repeated simple linear regression was performed to examine the association of a number of independent variables with gonorrhea incidence rates. Average HIV incidence rates, percentage of women, percentage of individuals of Black race, percentage of individuals under the poverty line and county urban ranking were positively associated with the incidence of gonorrhea (Table 2, all variables were population-adjusted).

**Table 2:** Univariable and multivariable linear regression analysis of *N*. *gonorrhoeae* infection rates at the county level.

	Univariable Analysis				Multivariable Analysis		
Variable	Regression Coefficient	95% Confidence Interval	R^2^	P	Regression Coefficient	95% Confidence Interval	P
**HIV incidence**	2.05	1.62, 2.48	0.40	<0.001	1.26	0.86, 1.66	<0.001
**Percentage of women**	0.73	0.52, 0.93	0.14	<0.001	0.41	0.28, 0.53	<0.001
**Urban ranking**	0.26	0.16, 0.37	0.03	<0.001	0.12	0.03, 0.20	0.005
**Poverty**	0.66	0.53, 0.79	0.33	<0.001	0.45	0.32, 0.57	<0.001
**Black race**	0.97	0.84, 1.09	0.60	<0.001	n/a	n/a	n/a

n/a: not applicable

Subsequently, the independent variables identified through the univariable analysis were combined to produce a multivariable linear regression model. HIV incidence rates, county urban ranking, percentage of women and percentage of individuals under the poverty line (Table 2) were independent predictors of gonorrhea incidence (Pmodel<0.001, R^2^ model = 0.56).

Notably, although Black race has previously been associated with increased risk of gonorrheal infection [8, 10, 11, 13, 20], we elected not to include race in the final model. This decision stemmed from the fact that race primarily reflects disparities in access to healthcare and the overall standard of living, as evidenced by the collinearity that race displayed with other demographic variables. In turn, this finding suggests that race could often simply function as a proxy of socioeconomic conditions, rather than a true independent risk factor and should be used with caution in epidemiologic studies [21, 22]. A patient-level analysis may be better suited to identifying the relative impact of the socioeconomic and biologic risk factors (such as absence of the CCR5 mutation in individuals of black race, leading to increased susceptibility to HIV [23–25]) that might underlie the association of race with the incidence of STIs.

Importantly, HIV incidence rates had a powerful association with *N*. *gonorrhoeae* infection. Sexually transmitted infections not only share common risk factors with HIV infection, but also facilitate HIV transmission [5, 26] and previous studies have reported that *N*. *gonorrhoeae* infection may result in up to a 3-fold increase in the risk of HIV seroconversion [6, 27, 28]. This is noteworthy, given the morbidity and healthcare costs associated with HIV infection. For example, a recent study estimated the lifetime cost of HIV infection at approximately $326,500 [29] and, as these figures were based on conservative assumptions, overall costs of HIV infection may be even higher in practice. Although previous trials that assessed the impact of treatment for STIs on HIV incidence produced inconclusive results [30, 31], some evidence suggests that timely therapy for STIs, including gonorrhea, may help to decrease HIV transmission, particularly in the setting of high STI and HIV incidence [32, 33].

Moreover, our findings emphasize the importance of preventive measures whenever an STI, such as gonorrhea, is diagnosed. For example, given the proven efficacy and relative safety of pre-exposure prophylaxis (PrEP) for HIV, health care providers should consider whether PrEP should be offered to patients with a history of an STI [34–36]. Recent recommendations of the International Antiviral Society-USA Panel suggest that PrEP should be discussed with patients that were recently diagnosed with an STI, particularly if their yearly risk of contracting HIV is 2% or more [34, 36]. Interestingly, a recent study by Solomon et al. suggested that Men who have Sex with Men and are diagnosed with syphilis are at very high risk for HIV seroconversion and may constitute ideal candidates for PrEP [37]. Although it may be tempting to hypothesize that an association may also exist between *N*. *gonorrhoeae* infection and subsequent HIV seroconversion, the present population-based study cannot establish causation or determine the nature and direction of the association between gonorrhea and HIV infection on an individual level. Patient-level analyses are needed to investigate the interplay of HIV and *N*. *gonorrhoeae* infection and determine the precise indications of PrEP administration in this patient group. Moreover, it must be noted that the decision to initiate PrEP does not obviate the need for patient education and appropriate counseling on sexual behavior, especially given the minimal cost and risk-free nature of such interventions [38].

This multivariable analysis also demonstrated that county poverty levels were independently associated with gonorrhea incidence rates. Socioeconomic disadvantage has been shown to exert an impact on *N*. *gonorrhoeae* infection [8, 11], and our results lend further credence to previous studies, by utilizing data on a national scale. Although the present analysis cannot conclusively demonstrate whether the reported association is directly causal, income status may serve as a proxy of community sexual health. Poverty is inextricably associated with low educational attainment [39], decreased access to health care [40], substance abuse [41, 42] and increased prevalence of prostitution [43], all of which are known to either promote high-risk sexual behavior or prevent the early identification and treatment of *N*. *gonorrhoeae* infection [10, 13]. Although some of these factors may appear challenging to address, positive changes in socioeconomic conditions may substantially impact gonorrhea incidence rates [11].

Lastly, our findings also point to increased gonorrhea incidence in urban counties, as well as in counties with a greater percentage of women. The first finding is consistent with the existing literature, which suggests that gonorrhea tends to be more frequent in the urban setting, particularly in areas characterized by increased population density and considerable socioeconomic disadvantage [10, 44]. Nevertheless, we must note that gonorrhea infection rates may be consistently underreported in rural areas [12]. As such, the importance of public health initiatives in the rural setting could be underestimated. Regarding the higher overall incidence of gonorrhea in counties with a larger female population, this finding may partly be attributed to the fact that women commonly develop asymptomatic infection [4]. In turn, although the rate of asymptomatic infection is not adequately captured by incidence statistics, asymptomatic individuals are central to disease transmission as they may unwittingly serve as a disease “reservoir”. In fact, mathematical models suggest that the presence of a preexisting high-incidence “reservoir” of *N*. *gonorrhea* infection in a sexual network may have a greater impact on disease spread than individual sexual behavior [45]. As such, our findings underlie the importance of targeted screening programs in high-risk women, as outlined in the recommendations recently issued by the U.S. Preventive Services Taskforce [46].

Regarding limitations that should be considered before interpreting our findings, the present analysis relied on aggregate incidence rates and sociodemographic data, collected at the county and state level, rather than data from individual cases. As such, detailed patient-level stratification according to possible confounders was impossible to perform. Furthermore, the cross-sectional design of the study precluded the determination of cause-and-effect relationships, but consideration of the existing literature facilitates interpretation of our findings. The databases we utilized enabled us to perform a nationwide analysis and limited the possibility of sampling bias. However, the timeframe of our analysis, as well as the availability of pertinent sociodemographic variables, were limited. Moreover, the possible existence of a systematic pattern of underreporting among counties with specific sociodemographic characteristics may have introduced bias into our analysis.

## Conclusions

In conclusion, the incidence of gonorrhea in the U.S. increased over the 2010–2014 time period. Given the high frequency of asymptomatic infection [4], the increasing rate of antibiotic resistance among *N*. *gonorrhoeae* strains [2] and the association of gonorrhea with HIV transmission [5], a comprehensive public health response is necessary to contain disease spread. We developed a county-level, multivariable linear regression model of *N*. *gonorrhoeae* infection, after identifying independent predictors of gonorrhea incidence on a national scale. Importantly, our analysis demonstrated that gonorrhea and HIV infection are closely associated at the population level. Although the present study cannot conclusively prove whether this association is also valid at the patient level, it suggests the potential benefits from comprehensive screening for STIs and HIV. This is particularly important, given the large number of unreported/undiagnosed STI cases [3, 47] and the low compliance with screening recommendations that is reported in the literature [48–51]. Furthermore, our findings highlight the potential value of offering pre-exposure prophylaxis for HIV to high-risk patients visiting an STI clinic, particularly in high-incidence areas. Sociodemographic factors were also associated with gonorrhea incidence, thus suggesting another possible focus for public health initiatives. Taken together, a combination of targeted screening programs, counseling on sexual behavior and comprehensive clinical management of high-risk patients with STIs may prove instrumental in curtailing the spread of gonorrhea and HIV infection. Future studies that focus on the most heavily affected areas will provide additional guidance on how to allocate scarce public health funds and design cost-effective initiatives.
